# Ameliorating Metabolic Profiles After Kidney Transplantation: A Protocol for an Open-Label, Prospective, Randomized, 3-Arm, Controlled Trial

**DOI:** 10.3389/fmed.2021.800872

**Published:** 2021-12-23

**Authors:** Saifu Yin, Ming Ma, Zhongli Huang, Yu Fan, Xianding Wang, Turun Song, Tao Lin

**Affiliations:** ^1^Department of Urology, West China Hospital, Sichuan University, Chengdu, China; ^2^Institute of Urology, West China Hospital, Sichuan University, Chengdu, China; ^3^Organ Transplant Center, West China Hospital, Sichuan University, Chengdu, China

**Keywords:** metformin, empagliflozin, kidney transplantation, metabolic disorders, clinical trial

## Abstract

**Aim:** High prevalence of metabolic disorders causes higher risk of cardiovascular diseases after kidney transplantation (KT), which remains the main burden impairing short-term and long-term survival. This open-label, prospective, randomized, 3-arm, controlled trial will evaluate the safety, tolerability and efficacy of metformin and empagliflozin in ameliorating metabolic profiles after KT.

**Methods:** After a screening assessment, eligible patients with an estimated glomerular filtration rate (eGFR) >45 mL/min/1.73m2 are randomly assigned to standard triple immunosuppression alone, standard immunosuppression plus metformin (500 mg twice daily), standard immunosuppression plus empagliflozin (25 mg once daily) from discharge. The primary endpoint is the differences in the visceral-to-subcutaneous fat area ratio over 12 months, evaluated by magnetic resonance imaging (MRI). Secondary outcomes include kidney graft function, glycometabolism, lipid metabolism, and inflammatory parameters. The trial will enroll 105 kidney transplant recipients, providing 90% power to detect the difference at 5% significance.

## Introduction

Advances in patient selection, organ procurement and preservation, surgical technique, immunosuppression, and infection prevention have conferred significant improvement in rejection, infection, and subsequently decreased cause-specific graft failure rates after kidney transplantation (KT) (1). However, cardiovascular diseases (CVD) remain the main burden impairing both short-and long-term patient survival (2). Compared with the general population, conventional CVD risk factors, including obesity, liver and muscle insulin resistance, dyslipidemia, hypertension, and diabetes mellitus, are all highly prevalent in this population for long-term exposure to steroids and calcineurin inhibitors (3–6).

Previous studies demonstrated that adenosine 5′-monophosphate (AMP)-activated protein kinase (AMPK) is a central regulator of multiple metabolic pathways and a key player in regulating cellular energy metabolism (7, 8). Activation of AMPK by pharmacological agents may hold a considerable potential to reverse the metabolic abnormalities in chronic metabolic diseases (9, 10). Metformin, a widely used antidiabetic drug, acts as an AMPK activator by inhibiting complex I of the mitochondrial electron transport chain in many tissues, including adipose, skeletal muscle, and heart (11). A recent small clinical trial observed that metformin administration can safely ameliorate metabolic profiles in glucocorticoid-treated patients with inflammatory disease but without pre-existing diabetes (12). In addition, another antidiabetic drug sodium-glucose-cotransporter-2 (SGLT-2) inhibitors can improve metabolic parameters and cardiovascular risk in patients with or without diabetes in pre-clinical and clinical studies (13, 14, 14, 15). Another small clinical trial even reported that compared to metformin, significant improvement in anthropometric parameters and body composition, in overweight and obese women with polycystic ovary syndrome after treatment with empagliflozin (16). Hence, metformin and SGLT2 inhibitors may be used as potential adjuvant therapies to improve metabolic disorders after KT.

Although several preliminary clinical trials showed that metformin and SGLT-2 inhibitors can be used safely and improve glucose control after KT, but they are small-sample sized and only include patients with diabetes (17–19). We will conduct a prospective clinical trial with the aim of exploring their roles in improving metabolic profiles.

## Methods and Analyses

### Study Design

**Figure 1:**
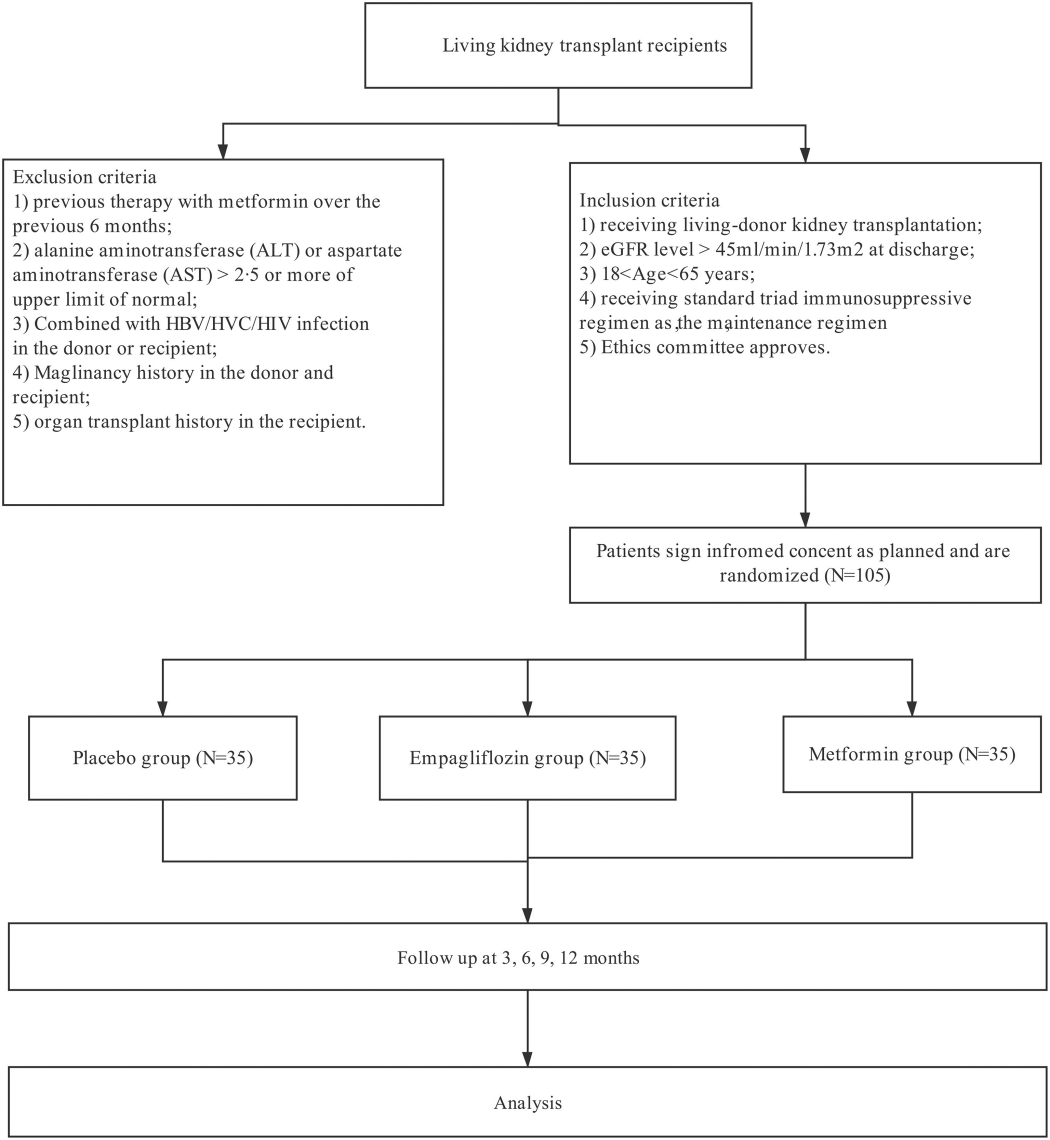
Patients selection.

This study is designed as a 12-month, single-center, prospective, 3-arm, open-label, randomized clinical trial (Figure 1). This study has been registered at www.clinicaltrials.gov (NCT05013112) and is conducted in accordance with the Declaration of Helsinki and local regulations. Selected patients are recruited if they meet the following inclusion criteria: (1) living-donor kidney transplantation; (2) eGFR level > 45 ml/min/1.73m2 at discharge; (3) 18 < Age <65 years; (4) receiving standard triad immunosuppressive regimen. Our key exclusion criteria are as follows: (1) previous therapy with metformin or SGLT 2 over the previous 3 months; (2) pre-transplant diabetes; (3) alanine aminotransferase (ALT) or aspartate aminotransferase (AST) > 2.5 or more of upper limit of normal; (4) Combined with HBV/HCV/HIV infection in the donor or recipient; (5) Malignancy history in the donor and recipient; (6) organ transplant history in the recipient. In addition, patients will exit from this clinical trial: (1) Serious deviation from clinical trial protocol; (2) Due to serious adverse reactions, the drug must be stopped in advance; (3) Withdrawal of informed consent; (4) Poor compliance and failure to use drugs as planned; (5) Lost to follow up.

### Randomization and Masking

Eligible participants are randomly allocated to standard immunosuppression (placebo group), standard immunosuppression plus metformin (metformin group), or standard immunosuppression plus Empagliflozin (Empagliflozin group). The randomization sequence is created using a random number generation function and allocation to each group is done through block randomization in a 1:1:1 ratio. The budesonide is open label.

### Procedures

Metformin is administered 500 mg twice daily orally from discharge based on the previous preliminary clinical trial (17). Empagliflozin is administered 25 mg once daily orally from discharge (18, 20). This will commence at randomization and continue for 12 months. The decision to continue with metformin beyond 12 months of follow-up is left to the discretion of the caring physician. In addition, during the follow-up, when kidney transplant recipients are diagnosed with PTDM, insulin therapy were adopted to reduce the glucose level at 6.1–6.7 mmol/L based on Chinese Management Guideline of post-transplantation diabetes mellitus after kidney transplantation (21).

Immunosuppressive regimen has been reported in our previous study (22). In brief, all participants will receive standardized triad immunosuppression, consisting of tacrolimus, mycophenolate mofetil/enteric-coated mycophenolate sodium, and steroids. Tacrolimus trough levels are measured by the enzyme multiplied immunoassay technique before breakfast and dose administration in the morning. Recommended tacrolimus trough levels are 5–10 ng/ml. MMF is started at 1000 mg one night before operation and maintained at 1000 mg twice daily with area under curve of mycophenolate mofetil maintained at 30–70 mg/h·L-1. If EC-MPS is selected, 720 mg is given the night before surgery and 720 mg bid thereafter. Methylprednisolone is injected intravenously at 500 mg during the operation and 200 mg per day 3 days after the operation. Then 60 mg prednisone is initiated, and gradually taper to 5–10 mg per day for maintenance.

### Data Collection and Clinical Outcomes

Standard demographic, clinical and laboratory data (including medication details) will be collected prospectively from electronic medical records. Recipients are advised to receive routine follow-up weekly in months 0–3, every 2 weeks in month 4–6, monthly within 6–12 months, and every 3 months thereafter.

The general aim of this study is to determine the safety of metformin and empagliflozin in kidney transplant recipients and explore the role of metformin and empagliflozin treatment in improving metabolic profiles. Based on previous study, visceral-to-subcutaneous fat area ratio, evaluated by magnetic resonance imaging (MRI), is generally reported as a surrogate for metabolic risk and is markedly raised in patients with long-term exposure to steroids (23, 24). Hence, the primary outcome is the differences in the visceral-to-subcutaneous fat area ratio over 12 months among three groups. The visceral-to-subcutaneous fat area ratio will be measured at transplantation, and post-transplant 3, 6, 9, and 12 months. Kidney graft function will be evaluated when they receive routine follow-up. Secondary outcomes included glycometabolism (fasting plasma glucose, fasting insulin levels, 2-h post-prandial insulin levels, hemoglobin A1c, and insulin beta-cell function [indicated by the homeostasis model assessment of β cell function]), lipid metabolism (serum triglyceride, non-high-density lipoprotein, low-density lipoprotein, total cholesterol), inflammatory parameters (C-reactive protein, interleukin-6, and tumor necrosis factor alpha) (Table 1). We will also examine cardiovascular disease and microvascular complications, including the following: major adverse cardiovascular events; all-cause mortality; atherosclerotic cardiovascular (ASCV) death; hospitalization or death from coronary artery disease, ischemic stroke, or heart failure; observable background diabetic retinopathy; and diabetic peripheral neuropathy.

**Table 1:** Routine follow-up.

**Visit**	**V0**	**V1**	**V2**	**V3**	**V4**
Time, days after kidney transplantation	−7day to 0 day	3 month	6 month	9 month	12 month
Participant-related information	×				
Informed consent	×				
History	×				
Inclusion/Exclusion	×				
Clinical examination	×	×	×	×	×
Efficacy and safety outcomes	×	×	×	×	×
Body weight	×	×	×	×	×
Height	×	×	×	×	×
Waist circumference	×	×	×	×	×
Visceral adipose tissue (VAT)	×	×	×	×	×
Subcutaneous adipose tissue (SAT)	×	×	×	×	×
VAT/SAT ratio	×	×	×	×	×
Glycometabolism	×	×	×	×	×
Fasting plasma glucose function	×	×	×	×	×
Fasting insulin levels	×	×	×	×	×
2-h postprandial insulin levels	×	×	×	×	×
Hemoglobin A1c	×	×	×	×	×
Insulin beta-cell function	×	×	×	×	×
Lipid metabolism	×	×	×	×	×
Serum triglyceride	×	×	×	×	×
Non-high-density lipoprotein	×	×	×	×	×
Low-density lipoprotein	×	×	×	×	×
Total cholesterol	×	×	×	×	×
Inflammatory parameters	×	×	×	×	×
C-reactive protein	×	×	×	×	×
Interleukin-6	×	×	×	×	×
Tumor necrosis factor alpha	×	×	×	×	×
Kidney graft function	×	×	×	×	×
Serum creatinine		×	×	×	×
Estimated glomerular filtration rate (eGFR)		×	×	×	×

### Sample Size Estimation

Sample size calculation is based on the primary outcome. According to the previous study, the visceral-to-subcutaneous adipose tissue area ratio was 0.83 (SD = 0.48; *n* = 58) for kidney transplant recipients (25). The ratio is estimated to be half of that in the intervention group. The estimated sample sizes for 90% power at 5% significance is 28 patients in each group. Considering an estimated 20% dropout rate, we assure that the sample size exceeds the minimal number needed to ensure the validity of the mean, effect size and rationale of feasibility. Therefore, 105 individuals (35 each group) are expected to be enrolled.

### Statistical Analysis

Two populations will be used in the analyses. The intention-to-treat (ITT) population will include all participants who have been randomly assigned, whereas the per-protocol (PP) population will include all participants who accomplish the entire intervention. The baseline characteristics of the study will be summarized as the means ± standard deviations for parametrically distributed data, geometric mean values (and 95% confidence intervals) for non-parametrically distributed data, and numbers (percentages) for categorical data.

Differences between participants who complete and withdraw from the trial will be analyzed by using a Student *t*-test or the Mann–Whitney test for continuous variables (e.g., age) and the chi-squared test for categorical variables (e.g., sex). For clinical outcomes, analysis of covariance will be used to examine differences among three groups, adjusting for potential confounding factors and effect modifiers (e.g., baseline age and sex). Based on the literature, some patients have higher risk of post-transplantation diabetes mellitus (PTDM), including those with family history of diabetes; or polycystic kidney disease; or age ≥60 years; or age 45–59 years plus (i) triglycerides ≥200 mg/dL; (ii) triglycerides 150–200 mg/dL and body mass index >27 kg/m2; (iii) triglycerides 150–200 mg/dL and high density lipoprotein (HDL) <40 mg/dL (men)/ <40 mg/dL (women) Hence, subgroup analysis will be conducted based on the risk of PTDM, and was also conducted based on the components of the criteria for high-risk PTDM (26–28). Our statistical analysis will be performed by using R software, and the results will be considered significant at a *P*-value of < 0.05.

## Discussion

This trial will provide more evidence on the safety and tolerability of metformin and SGLT2 agents in patients after renal transplantation. Most importantly, the study will also evaluate potential adjuvant therapies to ameliorating metabolic profiles in kidney recipients.

Successful developments in immunosuppression and clinical management make KT routines in adults with end-stage renal disease, but also unmask a greatly increased risk of premature metabolic disorders post-operatively. Generally accepted, long-term exposure to steroids can induce or worsen pre-existing insulin resistance, increase hepatic gluconeogenesis, and stimulate appetite and weight gain (5). In addition, calcineurin inhibitors can inhibit sterol 26–hydroxylase, diminish hepatic bile acid synthesis, reduce export of cholesterol from the liver, and inhibit β-cell growth and function (6). Compared with the general population, high prevalence of metabolic disorders increases the risk of post-transplant CVD, which makes CVD a main post-transplant burden. In Chadban's study including 23210 first kidney-only transplant recipients from 1980 through 2018 from the Australia and New Zealand Dialysis and Transplant Registry, the authors assessed the trend of all-cause and cause-specific mortality at different periods post-transplant (29). Although morality declined over successive eras at all periods, CVD remained the most common cause of death despite of reduced account from 50% in 1985–1989 to 30% in 2015–2018. Further analysis showed that in the current era (2015–2018), the adjusted death rates due to CVD was 0.28, 0.50, 0.96 per 100 patient-years with a follow-up of 1–5, 5–10, and >10 years. However, less attention is focused on how to improve metabolic profiles in this population.

Previous studies demonstrated that therapeutics aimed at activating AMPK remain promising and beneficial for improving metabolic disorders in the context of obesity, diabetes, cancer, non-alcoholic fatty liver disease, cardiovascular diseases (7–10, 30–32). In Kulkarni's study, they studied participants >70 years (*N* = 14) in a randomized, double-blind, placebo-controlled, crossover trial in which they were treated with 6 weeks each of metformin and placebo (33). The authors observed that both metabolic and non-metabolic pathways were significantly influenced, including pyruvate metabolism, mitochondrial fatty acid oxidation, and collagen trimerization in adipose. Similar improvements of metabolic disorders were also reported in those patients diagnosed with cancer and treated by metformin in a well-designed clinical trial (34). Another recent clinical trial reported that metformin can improve metabolic profiles in Cushing's syndrome without diabetes and receiving systematic steroids ((≥20 mg/day for ≥4 weeks and remaining on ≥10 mg/day for the subsequent 12 weeks, or its cumulative dose-equivalent) (12). 53 patients were randomly assigned to receive either metformin (*n* = 26) or placebo (*n* = 27) for 12 weeks. Improvements in markers of carbohydrate, lipid, liver, and bone metabolism were observed in the metformin group compared with the placebo group. These studies demonstrated available benefits in metabolic profiles for patients without diabetes.

However, metformin was also associated with potential side effects, such as metformin-associated lactic acidosis (35). Metformin plasma concentrations are ~2–4 folds higher in patients with type 2 diabetes and moderate to severe renal impairment (eGFR of 30 to <60 mL/min/1.73 m2 or <30 mL/min/1.73 m2, respectively) compared to healthy subjects (36). In a community-based cohort study, 75,413 patients with diabetes and time-dependent assessment of eGFR stage from January 2004 until January 2017 were included (37). The authors reported that there were 2335 hospitalizations with acidosis over a median follow-up of 5.7 years. Further analysis demonstrated that time-dependent metformin use was associated with incident acidosis only in patients with eGFR <30 mL/min/1.73m2 (HR: 2.07; 95% CI: 1.33–3.22), but not in patients with eGFR 45–59 mL/min/1.73m2 (HR: 1.16; 95% CI: 0.95–1.41) and eGFR 30 to 44 mL/min/1.73m2 (HR: 1.09; 95% CI: 0.83–1.44). This showed that metformin therapy may be safe in patients with eGFR 30–60 mL/min/1.73m2. For kidney transplant recipients, evidence of metformin is insufficient. A recent small clinical trial demonstrated that metformin can be administered safely in transplant recipients with stable kidney graft function but impaired glucose tolerance (*N* = 19) (17). Hence, considering potential benefits of metformin, it is time to reassess the safety and value of metformin in kidney transplant patients.

Another first-line anti-diabetic drug, SGLT2 had definite cardiovascular and renal function protection in patients with or without diabetes reported by many large clinical trials (13–15). In addition to improving glycometabolism, large clinical trials showed that SGLT-2 inhibitors can improve metabolic parameters in patients with or without diabetes (14, 38). Also, this action mechanism is insulin-independent; as such it does not increase the risk of hypoglycemia, making it attractive for use in normoglycemic individuals (39). Davies et al. demonstrated that assessed the effect of canagliflozin on the components of metabolic syndrome of metabolic syndrome in patients with T2DM and metabolic syndrome (38). The authors indicated that canagliflozin can significantly reduce HbAc1, fasting plasma glucose, body weight, body mass index, waist circumference, blood pressure, and triglycerides, and increased high-density lipoprotein cholesterol, and low-density lipoprotein cholesterol. Similar improvement of metabolic parameters was also seen in patients without diabetes. In the small clinical trial by Javed et al. the authors evaluated the effects of empagliflozin on metabolic parameters in polycystic ovary syndrome (16). Women with polycystic ovary syndrome were randomized to either empagliflozin 25 mg (*n* = 19) or metformin 1500 mg (*n* = 20) daily for 12 weeks with the main outcomes as the changes in anthropometric and body composition, and metabolic parameters. Despite of no statistical difference in metabolic parameters between two groups, there was a significant improvement in anthropometric parameters and body composition, in patients receiving empagliflozin.

However, SGLT2 agents are also restricted in kidney transplant recipients due to their mechanism of elimination and action by concentrate glucose in the urine. This can increase risk of urinary tract infections (40, 41), especially in patients with immunosuppression. Two recent small clinical trials have evaluated the safety and efficacy of SGLT2 in treating diabetes after kidney transplantation (18, 19). In the trial by Halden, 22 renal transplant recipients with diabetes were randomized to receive 10 mg empagliflozin with 20 receiving placebo as the control group once daily for 24 weeks. The authors shows that empagliflozin appeared safe and improved glycemic control in renal transplant recipients with NODAT compared with placebo. Also, a concomitant reduction in body weight was seen (443). In another preliminary prospective interventional trial including 14 kidney transplant recipients with NODAT and receiving insulin, the authors showed that empagliflozin can safely be used as add-on therapy when monitored closely (454). The latest clinical trial even showed that dapagliflozin can improve kidney function and reduce the risk of end-stage kidney disease and cardiovascular death in patients with stage 4 CKD and albuminuria (42). Hence, prospective studies are needed to explore the safety, tolerability and efficacy of metformin in improving metabolic profiles kidney transplant recipients with or without diabetes.

The limitation is that this study will explore the short-term impact of the intervention because the follow-up is set at 1 year. However, the development of metabolic diseases is a long and gradual process and can be influenced by other factors.

In summary, the AMPKT study will be the first dedicated clinical trial to explore the potential benefits and risks of metformin and SGLT2 inhibitors in kidney transplant recipients both with and without diabetes.

## Ethics Statement

The studies involving human participants were reviewed and approved by the Ethics Committee of West China Hospital. Written informed consent was not provided because this is a protocol for a Clinical Trial.

## Author Contributions

SY, XW, TL, and TS were the initiators of this study. SY and MM wrote this protocol. SY, XW, TS, ZH, YF, and TL tool part in the study planning. SY and XW wrote the final manuscript. All authors have read and approved the final manuscript.

## Funding

This work was supported by grants from the National Natural Science Foundation of China [Grant Number 81870513]; Sichuan Science and Technology Program [Grant Number 2019YJ0133]; Chengdu Science and Technology Program [Grant Number 2019-YF05-00084-SN]; and 1.3.5 Project for Disciplines of Excellence-Clinical Research Incubation Project, West China Hospital, Sichuan University [Grant Numbers 2018HXFH049, ZYJC18004, ZY2016104, and 2021HXFH007]. The funders had no role in study design, data collection or analysis, preparation of the manuscript, or the decision to publish.

## Conflict of Interest

The authors declare that the research was conducted in the absence of any commercial or financial relationships that could be construed as a potential conflict of interest.

## Publisher's Note

All claims expressed in this article are solely those of the authors and do not necessarily represent those of their affiliated organizations, or those of the publisher, the editors and the reviewers. Any product that may be evaluated in this article, or claim that may be made by its manufacturer, is not guaranteed or endorsed by the publisher.
